# Hospital Meetings

**Published:** 1905-01-21

**Authors:** 


### HOSPITAL MEETINGS.

### THE ROYAL WATERLOO HOSPITAL FOR CHILDREN AND WOMEN.

THE Lord Mayor (Mr. Alderman J. Pound) presided over the 89th Annual Court of the Royal Waterloo Hospital, held at the Mansion House on Tuesday, 17th inst, when a number of friends of the institution were present. The chairman was supported by Sir Edwin Durning-Lawrence, chairman of the hospital, Mr. J. Topham Richardson, treasurer, Sir George Hayter Chubb, and others.

The report for 1904 having been submitted by the secretary (Captain Houston), the Lord Mayor moved "That the Royal Waterloo Hospital for Children and Women is in every way worthy of support, and has strong and peculiar claims upon the City of London." The hospital, the Lord Mayor said, was one of the oldest in London, and a succession of Lord Mayors had held the office of president which, in his official capacity, now devolved upon him. This, he thought, constituted an undeniable claim on the support of the City. He rejoiced in the progress of the work of reconstruction, and trusted that an additional number of beds would shortly be opened. When the extent and value of the efforts for the relief of the suffering children and women of a very poor district, and the peculiar position occupied by the hospital, on the south side of the river, were taken into consideration, he thought sympathy and practical support could hardly be refused That the hospital had merited the approval of the Hospital Sunday Fund was in itself a strong recommendation since that Fund was administered by "past masters" in the art of hospital administration.

In moving the adoption of the report and accounts, the Treasurer said the total ordinary income for 1904 was £2,474 19s. 5d., as compared with £1,607 19s. 7d. in the previous year, and that legacies to the amount of £2,155 had been received. He assured the subscribers and other friends of the hospital that every item of expenditure was most carefully watched by those responsible for the administration of the funds. The cost of the portion to be opened, providing 100 beds, would be about £33,000, and towards this about £19,000 was in hand, leaving £14,000 to be raised. It was not proposed to proceed further until the way was clear to meeting the expense, and a special appeal was now being organised, upon which great hopes were based. The report and accounts having been adopted, Mr. Topham Richardson further moved—

That inasmuch as the present income is quite insufficient for the maintenance of the new hospital, and the building fund is already largely overdrawn, there is a most urgent need for new annual subscriptions, and for donations to the building fund.

The resolution was carried unanimously.

Sir Edwin Durning-Lawrence proposed a resolution, to the effect that the Court welcomed the formation of the Ladies' Association. It was now intended, he said, to start a Gentlemen's Association, since it was felt that the work of raising funds must not be left to the ladies.

Sir Geo. Hayter Chubb replied on behalf of the Ladies' Association, remarking that the Ladies' Committee, of which the association was a reconstitution, had in a few months raised £4,500. A Children's Guild, of which H.R.H. Princess Alexandra of Teck had undertaken the presidency, was in process of formation, and it was felt that the children of the wealthy could not more appropriately help the poor and sick of their own age than by becoming members.

A vote of thanks to the Lord Mayor and the Lady Mayoress and the Sheriffs having been passed, it was announced that a gigantic Christmas tree was being prepared for February 2nd at the Kensington Town Hall, when each child present would receive at least one present, the proceeds of the sale of tickets being devoted to the endowment of cots in the hospital.

